# Personalized Medicine in Autoimmune Diseases

**DOI:** 10.3390/jpm11111181

**Published:** 2021-11-11

**Authors:** Roberto Díaz-Peña

**Affiliations:** 1 Liquid Biopsy Analysis Unit, Oncomet, Health Research Institute of Santiago (IDIS), 15706 Santiago de Compostela, Spain; roberto.diaz.pena@sergas.es; 2 Faculty of Health Sciences, Universidad Autónoma de Chile, Talca 3460000, Chile

Autoimmune diseases are multifactorial disorders caused by both genetic and environmental factors and without a known cure. In these diseases, different treatment approaches may be employed to achieve remission or at least attenuate the symptoms. For personalized medicine to become a reality, a clear identification of groups of patients relatively homogenous, sharing pathogenic signaling pathways, is needed. For this reason, research related to autoimmune diseases is mainly focused on the identification of novel biomarkers, the discovery of new therapeutic targets and agents, and the elucidation of the mechanisms related to the etiopathogenesis of the different types of disease. The reality is that we are in the very early stages of personalized medicine for autoimmune diseases.

A good example of how the search for biomarkers may help us to manage patients in a personalized way is found in oncology, where there are a large number of molecular tests for most metastatic tumors to guide treatment decisions. However, the continued implementation of precision oncology is largely due to the presence of strong genetic drivers of disease that is enabling the classification of disease heterogeneity, missing for most autoimmune and inflammatory diseases. Here, the underlying genetics are highly complex, and the effect of individual genetic markers has a modest contribution to disease. In this regard, Díaz-Peña et al. reviewed current findings on the genetics of spondyloarthropathies (SpA) [1], a group of chronic inflammatory diseases highly heritable. The development of applications from genome-wide association studies (GWAS) data, such as polygenic risk scores (PRS), seems to have a potential value in SpA diagnosis and even in prognosis and response to treatment.

The implementation of personalized medicine depends on the processing of the information obtained at a group level population, and its application to the specific biological, genetic, and clinical characteristics of an individual. However, there are other factors that provide additional complexity and need to be considered, such as ethnicity, lifestyle differences, and environmental factors. In this context, a relevant gap between Caucasian and non-Caucasian target populations exists, necessary to overcome for the implementation of personalized medicine in all populations. In this regard, Vetchinkina et al. identified genetic variants associated with demographic and clinical characteristics of rheumatoid arthritis (RA) patients in the Russian population [2]. The article adds to the scientific literature on RA genetics by testing a number of known risk factors in an ethnic group not tested before. Díaz-Peña et al. investigated the contribution of environmentally established risk factors, genetic ancestry, single nucleotide polymorphisms (SNPs), and their association with chronic obstructive pulmonary disease (COPD) risk in a Latin American population, characterized by a large admixture of Amerindian ancestry [3]. An association between the PPP1R12B gene and COPD, as well as a significant effect modification between ethnicity and PRDM15 gene variants in determining COPD risk, was reported. In addition to these basic scientific papers, one review article presented the latest advances in our understanding of genetic determinants of RA risk among underrepresented populations [4]. Advances in GWAS have significantly improved our understanding of mechanisms underlying the development of autoimmune diseases. However, there is a small proportion of Africans and Latin American people included in GWAS. This loss of genomic diversity in genetic studies may decrease the capacity for benefits obtained in other populations as a result of the increased knowledge. The next few years will witness breakthroughs in personalized medicine, providing keys for the prediction of response to treatment and/or disease severity and/or prognosis in autoimmune diseases, and not all knowledge may be extrapolated from the Caucasian data. Results highlight the importance to conduct more GWAS in underrepresented populations.

Unfortunately, for most autoimmune diseases, the heterogeneity of the patients causes a non-uniform response to a given therapy. Three review articles address this issue with different approaches. Ghiboud et al. summarized the most recent advances in the development of inhibitors that present a high degree of selectivity in targeting the epigenetic modulators [5], specifically the histone deacetylases (HDACs) inhibitors and bromodomain and extraterminal (BET) inhibitors, and their potential to be used in autoimmune and inflammatory diseases. Further, corticosteroids are particularly effective against most autoimmune diseases. Son et al. examined clinical outcomes of corticosteroid treatment in patients with sepsis or septic shock [6]. The authors concluded that the combination of hydrocortisone, fludrocortisone, and dexamethasone can be a promising therapeutic option to improve survival outcomes in sepsis patients. Finally, Mazini et al. presented a review focusing on the relationships between the adipose-derived stem cells (ADSCs) and their microenvironment [7], involving different pathways and potential targets that might serve as specific biomarkers of their immunomodulating capacity in skin regeneration and aging.

Two papers discussed the potential of plasma biomarkers. Bure et al. analyzed the capability of microRNAs (miRNAs) as predictive factors for the clinical response to therapies among RA patients [8]. The authors revealed statistically significant associations with the olokizumab therapy efficiency scores for miR-26b, miR-29, miR-451, and miR-522 levels, suggesting these miRNAs might be a potential therapeutic response biomarker. Mondelo-Macía et al. summarized the basic biology of circulating free DNA (cfDNA) including their clinical interest in inflammation and autoimmunity [9]. It is proposed that cfDNA may be a useful biomarker of disease activity, prediction of the therapeutic response, and progression in autoimmune diseases, at least in systemic lupus erythematosus (SLE) and RA.

Finally, Rioseras et al. analyzed the adhesion and migration abilities of CD4+CD28null T lymphocytes in RA patients, as well as the effect that IL-15 could have in these processes [10]. CD4+CD28null T lymphocytes, increased in RA patients, showed enhanced migration capacity in comparison with their counterparts CD28+ T lymphocytes. IL-15 exerted a potential effect on this migration ability, especially in CD4+CD28null T lymphocytes. The authors identified a list of genes and proteins related to cell migration pathways that could be involved in CD4+CD28null lymphocytes’ migration to the target tissue, where they promote the inflammatory damage. The potential biomarkers described could be investigated in the future as therapeutic targets in RA patients.

The next years will deliver significant advancement in next-generation sequencing techniques and in statistical approaches, allowing us the integration of multiple “-omics”, and thus to improve the current knowledge on complex diseases, autoimmune diseases in this case. Articles in this Special Issue offer substantial and specific information about a broad range of topics around personalized medicine in autoimmune diseases that will generate impact in the near future, and above all, represent a taste of what remains to be accomplished.

## Data Availability

Not applicable.
